# Human-Centric AI: The Symbiosis of Human and Artificial Intelligence

**DOI:** 10.3390/e23030332

**Published:** 2021-03-11

**Authors:** Davor Horvatić, Tomislav Lipic

**Affiliations:** 1 Department of Physics, Faculty of Science, University of Zagreb, Bijenička Cesta 32, 10000 Zagreb, Croatia; 2 Division of Electronics, Ruđer Bošković Institute, Bijenička Cesta 54, 10000 Zagreb, Croatia

**Keywords:** artificial intelligence, deep neural networks, interpretability, explainability, fairness, accountability, transparency, human-centric AI, human-like learning, intelligence augmentation

## 1. Introduction

Well-evidenced advances of data-driven complex machine learning approaches emerging within the so-called second wave of artificial intelligence (AI) fostered the exploration of possible AI applications in various domains and aspects of human life, practices, and society. Most of the recent success in AI comes from the utilization of representation learning with end-to-end trained deep neural network models in tasks such as image, text, and speech recognition or strategic board and video games. By enabling automatic feature engineering, deep learning models significantly reduce the reliance on domain-expert knowledge, outperforming traditional methods based on handcrafted feature engineering and achieving performance that equals or even supersedes humans in some respects.

Despite the outstanding advancements and potential benefits, the concerns about the black-box nature and the lack of transparency behind the behavior of deep learning based AI solutions have hampered their further applications in our society. To fully trust, accept, and adopt newly emerging AI solutions in our everyday lives and practices, we need human-centric explainable AI (HC-XAI) that can provide human-understandable interpretations for their algorithmic behavior and outcomes—consequently enabling us to control and continuously improve their performance, robustness, fairness, accountability, transparency, and explainability throughout the entire lifecycle of AI applications. Following this motivation, the recently emerging trend within diverse and multidisciplinary research communities is based on the exploration of human-centric AI approaches and the development of contextual explanatory models propelling the symbiosis of human intelligence (HI) and artificial intelligence (AI), which forms the basis of the next (third) wave of AI.

## 2. Themes of This Special Issue

This Special Issue aims at collecting original and high-quality papers focusing on methodologies, techniques, and tools for achieving explainability in different complex machine learning models, their outputs, and their behaviors (e.g., directly by self-explainable, intrinsically interpretable models, through learning disentangled representations, post hoc local interpretability, explanations via examples), which are intended for specific target users (e.g., machine learning experts, domain experts, and general end-users) and are evaluated on different synthetic or real-world datasets and settings coming from various disciplines.

The current approaches based on information-theoretic principles mostly focus on an aspect of advancing algorithmic transparency and understanding the learning dynamics of complex machine learning algorithms (e.g., see contributions to other related Entropy Special Issues [1,2]). Therefore, we encouraged multi-disciplinary researchers to explore additional aspects of interpretability and novel approaches for contextual explanatory models through the lens of information theory, statistical physics, and complexity science in general. Contributions were invited considering these aspects, including:

- Information theoretic principles on the connection between accuracy, reliability, robustness, fairness, accountability, transparency, or explainability in different types of deep learning models.
- Methodologies and tools providing global or local human-interpretable explanations for predictions of general or specific complex machine learning and deep learning models for dealing with time series, sequences, graph structured and heterogeneous relational data, or other specifically structured data, as well as different types of unstructured data. Information-theoretic- and complexity-based techniques and metrics to assess the quality of the explanations.
- Methodologies and tools for understanding learned distributed representations, information flows, and algorithmic transparency in different deep learning network models and architectures with a specific focus on the information theory and statistical physics aspects.
- Case studies utilizing explainable AI and machine learning approaches for scientific discoveries in various disciplines such as computational biology, biophysics, medicine, neuroscience, social science, or digital art and humanities.
- Case studies and techniques for providing human-like concept learning in AI systems by exploring and utilizing principles of causality, learning-to-learn (meta-learning), one/few-shot, incremental, and active machine learning.

The foundation for making the symbiosis of human and artificial intelligence feasible is to provide human-centric explainable AI. We can explore this human-centered partnership of people and AI from two perspectives: (1) intelligence augmentation and the (2) Human Intelligence (HI) for Artificial Intelligence (AI) perspective (HI4AI). After review, a total of 10 papers have been accepted for publication in this issue, which can be roughly grouped in contributions with respect to these two conceptual perspectives of the symbiosis of human and artificial intelligence. The first article of this Special Issue was published on 18 August 2020, and the last on 25 January 2021. The articles are briefly discussed below.

### 2.1. Contributions to Intelligence Augmentation

At one end, AI provides an assistive role in advancing human capabilities and cognitive performance, commonly referred to as augmented intelligence. In general, the goal is to improve the efficiency of the human decision-making process using automation complemented with human reasoning to manage the potential risks of automated decisions.

Most of the included papers are application-based systems covering inspiring case studies of utilizing Human-Centric explainable AI and machine learning approaches in safety-critical applications such as medical imaging and credit risk modeling or in scientific discoveries of physics phenomena. When adopting deep learning models in such scenarios, we need to have interpretable and robust explanations for their decisions and behavior and cannot only rely on good prediction performance.

In the contribution by Jin et al. [3], “Optic Disc Segmentation Using Attention-Based U-Net and the Improved Cross-Entropy Convolutional Neural Network,” following the trend of successful application of channel attention mechanism in the field of medical image segmentation, the authors proposed novel aggregation channel attention upsampling module to make full use of the influence of context information on semantic segmentation by exploiting channel dependencies and integrating information of different scales into the attention mechanism. Experimental results showed that utilizing this attention mechanism has a good effect on the task of optic disc segmentation of fundus images while balancing the contribution of dice coefficients and cross-entropy loss to the segmentation task enhanced performance in small area segmentation.

Another contribution by Barić et al. [4], “Benchmarking Attention-Based Interpretability of Deep Learning in Multivariate Time Series Predictions,” sets the newly emerging trend of specifically designing diagnostic datasets for understanding the inner workings of attention mechanism based deep learning models for multivariate forecasting tasks. The authors designed a novel benchmark of synthetically designed datasets with the transparent underlying generating process of multiple time series interactions with increasing complexity. Experimental results based on artificially created transparent benchmark datasets revealed insightful weaknesses in current state-of-the-art, attention-based architectures’ performance and behavior. Most models exposed satisfying and stable prediction performance results. However, they often failed to give correct interpretability, which interestingly increases with the complexity of interactions between multiple time series (i.e., the harder the task, the more correct is a model in interpreting its decisions).

In the contribution by Merćep et al. [5], “Deep Neural Networks for Behavioral Credit Rating,” the authors proposed benchmarking setting for evaluating the potential of deep learning for predicting probabilities of default (PD) in a portfolio of loans, to stand in as nonlinear methodology and alternative to logistic regression, which is currently the long-established industry standard in credit risk modeling due to regulatory requirements for the explainability of the model output. The deep learning model for behavioral credit risk assessment displayed significant performance improvement compared to linear benchmark models. However, it was evenly matched with another nonlinear, more intrinsically interpretable, the XGBoost model. The presented study gives inspiring use cases and reveals the need to further develop HC XAI methodology to completely satisfy the regulatory requirements for model explainability.

The contribution by Mohr et al. [6], “Predicting the Critical Number of Layers for Hierarchical Support Vector Regression,” gives an inspiring example of how complex system analysis can facilitate learning dynamics transparency of modern nonlinear machine learning algorithms in order to better understand the optimal way of their development and application. Specifically, the authors proposed a novel hyperparameter optimization technique that determines the critical number of layers in a hierarchical support vector machine (HSVM) from time-series data. The proposed method adopts two approaches—Fourier Transform (FT) and Dynamic Mode Decomposition (DMD)—to predict the sufficient number of layers required to achieve the given value of the model error threshold. The analysis showed that utilization of the DMD approach gives more efficient models having less layers, while determining hyperparameters guided with FFT gives models with smaller errors but more layers.

Jercic and Poljak [7], in their contribution titled “Exploring the Possibility of a Recovery of Physics Process Properties from a Neural Network Model,” describe a procedure to investigate the capability of a Neural Network to extract information about the underlying physics behind data collected by particle accelerators, such as the Large Hadron Collider (LHC) at CERN. Specifically, the authors test the whole procedure by “imposing” a given fragmentation statistic, produce phantom data, blind analyze these data via a convolution neural network, and compare the determined probability distribution with the imposed one, showing that some characteristics of the physical process can be retrieved with the help of neural networks.

In the contribution by Grubišić et al. [8], “Deep Neural Network Model for Approximating Eigenmodes Localized by a Confining Potential,” a class of physics-informed deep dense neural networks is used to learn the mapping from potential to ground eigenstate for the Schrödinger equation for several confining potentials. The authors present an approach that combines the expressivity of the set of neural network realizations with the standard error indicators. The approach has the potential to lead to robust approximation methods.

### 2.2. Contributions to HI4AI Perspective—Human Intelligence (HI) for Artificial Intelligence (AI)

Human intelligence can serve either as information feedback with a human in the loop to usefully inform the processes of AI development, deployment, and operation, or serve as an inspiration for novel design principles based on reverse engineering human intelligence (e.g., human-like concept learning, progressive learning, creativity, general-purpose reasoning).

The contribution by Kulikovskikh et al. [9], “From Knowledge Transmission to Knowledge Construction: A Step towards Human-Like Active Learning,” explains how deep learning models to decisions by imitating human-like reasoning in multiple-choice testing. The authors proposed a new strategy that measures the information capacity of data using the information function from the four-parameter logistic item response theory (4PL IRT) and compared the proposed strategy (termed Information Capacity) with the most common active learning strategies—Least Confidence and Entropy Sampling. The computational experiments results showed that the Information Capacity strategy shares similar behavior but provides a more flexible framework for building transparent knowledge models in deep learning. By considering an active learning environment in an educational setting, this is the only contribution that addressed the perspective of utilizing human intelligence for artificial intelligence (HI4AI) from both aspects, namely the human in the loop aspect (by exploring active learning conceptual framework) and the human-inspired intelligence (i.e., reverse engineering HI) aspect (by imitating human-like reasoning in multiple-choice testing).

The contribution by Song [10], “Personalized Image Classification by Semantic Embedding and Active Learning,” proposed utilizing an active learning algorithm to dynamically select which images to be annotated or verified in order to improve the efficiency of a novel interactive system for personalized image classification. Conducted experiments show that the system provides a flexible and efficient tool for user-adaptive image classification, focusing on personalized classification with different granulates. The presented study gives an uplifting example of the need for a mutualistic symbiosis of human intelligence (HI) and artificial intelligence (AI).

In the contribution by Zhang [11], “Visual Speech Recognition with Lightweight Psychologically Motivated Gabor Features,” the authors were inspired by human-centric glimpse-based psychological research of how humans recognize faces, designed a novel efficient deep learning based speech recognition system. This is an amazing example of how the development of novel, more understandable, and efficient deep learning based AI systems (that could also learn from less data) can be promoted by human-like concepts explored in different multidisciplinary fields.

Finally, Unceta, Nin, and Pujol [12], in their contribution entitled “Environmental Adaptation and Differential Replication in Machine Learning,” were inspired by theoretical concepts (differential replication) and framework grounded in biological evolution in order to solve what they term the environmental adaptation problem of machine learning models. In this newly introduced machine learning setting, as opposed to transfer learning and domain adaptation, a change in the conditions of a model demands the definition of a new feasible set of solutions because the solution in the source scenario is unfeasible in the target scenario. The authors proposed differential replication of machine learning models, enabling “model survival in highly demanding environments” based on the knowledge acquired by previously trained models in generations. This nature-inspired approach gives novel conceptual thinking for the development of machine learning based AI systems.

## 3. Conclusions

The articles presented in this Special Issue provide insights. We thank all the authors for their excellent contributions and timely submission of their works. We are looking forward to many future developments that will build on the current bounty of insightful results and that will explain AI systems better.
